# The Role of Mirror Therapy in the Rehabilitation of the Upper Limb’s Motor Deficits After Stroke: Narrative Review

**DOI:** 10.3390/jcm13247808

**Published:** 2024-12-20

**Authors:** Ioannis Ventoulis, Kyriaki-Rafaela Gkouma, Soultana Ventouli, Effie Polyzogopoulou

**Affiliations:** 1 Department of Occupational Therapy, University of Western Macedonia, Keptse Area, 50200 Ptolemaida, Greece; kyriaki.gkouma02@gmail.com; 2 Department of Statistics and Insurance Science, University of Western Macedonia, 6th km of Old National Motorway Grevena-Kozani, 51100 Grevena, Greece; sgventouli@gmail.com; 3 Emergency Medicine Department, Attikon University Hospital, National and Kapodistrian University of Athens, Rimini 1, Chaidari, 12462 Athens, Greece; effiepol@med.uoa.gr

**Keywords:** mirror therapy, stroke, rehabilitation, neurorehabilitation techniques, motor function, motor impairment, motor deficits, upper limb

## Abstract

Stroke is one of the leading causes of death and disability worldwide and poses a tremendous socioeconomic burden upon individuals, countries and healthcare systems. It causes debilitating symptoms and thus interferes with many aspects of the patient’s life, including physical functioning, cognition, emotional status, activities of daily living, social reintegration and quality of life. Post-stroke patients frequently experience functional motor disabilities of the upper limb, which restrict autonomy and self-efficacy and cause limitations in engagement with activities and social participation, as well as difficulties in performing important occupations. It is therefore not surprising that motor impairment or loss of motor function of the upper limb is one of the most devastating sequelae of stroke. On these grounds, achieving optimal functioning of the upper limb after stroke remains a fundamental goal of stroke rehabilitation. Mirror therapy (MT) represents one of the several rehabilitation techniques used for restoring the upper limb’s motor function after a stroke. However, conflicting results about the role of MT in the rehabilitation of the upper limb’s motor deficits have been reported in the literature. Accordingly, the aim of this narrative review is to summarize existing evidence regarding the effects of MT on the upper limb’s motor function in post-stroke patients and to further explore its role when applied in different phases of stroke.

## 1. Introduction

Despite recent advances in the treatment and prevention of stroke, stroke continues to constitute a global epidemic with high morbidity and mortality rates [1]. According to the Global Burden of Disease (GBD) Study 2021, stroke is the third leading cause of death, accounting for 7.3 million deaths worldwide, and the fourth most common cause of disability-adjusted life-years (DALYs) [2]. Undoubtedly, stroke poses a huge economic burden on healthcare systems and societies, considering the fact that an individual with stroke requires inpatient care, rehabilitation and lifelong follow-up, let alone the loss of independence and productivity incurred by the long-term functional, cognitive and psychological sequelae of stroke [3,4].

Indeed, stroke often results in loss or impairment of the motor control and function of the upper limb, which immensely affects activities of daily living and quality of life. Motor impairment of the upper limb after stroke poses a significant challenge for healthcare providers involved in the process of stroke rehabilitation [5,6,7]. In order to address the issue of functional impairment and improve motor function of the paretic upper limb, several rehabilitation techniques have been introduced, most of which are mainly applied to patients with mild or moderate impairment of the upper limb [8,9]. To cover this gap, mirror therapy (MT) has been proposed as a potentially beneficial alternative method in studies involving patients with severe or complete motor deficits of the upper arm [10,11,12].

The application of MT in patients with stroke is based on the following fundamental principle: Through the use of a mirror, MT utilizes the creation of an optical illusion and the generation of real-time visual feedback as a rehabilitation technique aiming at retraining patients with hemiparesis, restoring motor function of the hemiparetic limb and improving functional disability. During this method, a mirror is placed in the mid-sagittal plane of the patient’s torso between the two extremities and the patient is instructed to sit comfortably and place their limbs at each side of the mirror, so that the paretic limb lies behind the mirror (on the mirror’s non-reflective side) and the unaffected limb lies in front of the reflective side of the mirror. By accommodating this position, the paretic arm remains hidden from the patient’s visual field and the patient can only view the unaffected limb, as well as the reflected image of the unaffected limb on the mirror, which is superimposed in the perceived position of the paretic arm, thereby creating a visual illusion that the reflected image is actually the paretic arm. The patient is initially asked to practice imagined bilateral movements, first with the unaffected and then with the affected limb. Once the patient becomes familiar with this mental task of imagined movements, they are then asked to observe the reflection of the unaffected limb in the mirror without moving it yet and try to get engaged with the mirrored limb as if it were the paretic one. After having completed this preliminary stage, the patient is subsequently instructed to perform movements with their unaffected extremity, while observing its reflected image on the mirror as if it were the affected extremity. By doing so, the movements of the unaffected limb are superimposed on the mirror and create an optical illusion that their paretic limb is also executing the same normal movements. The aforementioned task may involve either unilateral movements of the unaffected limb only or bilateral movements of both the affected and unaffected limbs [13,14].

**Figure 1:**
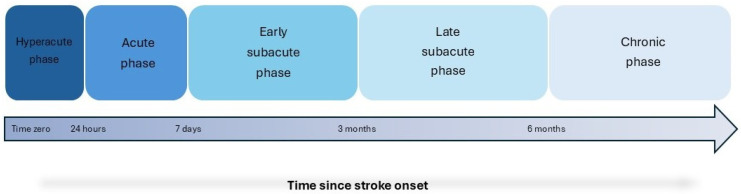
Timeframe of stroke recovery.

MT has been widely used in clinical practice by scientists involved in the rehabilitation process of patients with motor deficits of the upper limb due to stroke. MT has been applied in different stages of stroke, in various clinical settings and by using diverse therapeutic protocols [15,16,17]. Accordingly, the aim of this narrative review is to summarize existing evidence regarding the effects of MT on the upper limb’s motor function in post-stroke patients and to further explore its role when applied in different phases of stroke. For the purpose of this narrative review, we searched for relevant articles via PubMed (MEDLINE) database using a combination of keywords, such as “mirror therapy”, “stroke”, “upper limb/extremity/arm”, “motor function”, “motor deficits/impairment”, “rehabilitation”. The cited bibliography of the retrieved articles was also searched for further relevant sources. Relevant MT studies will be presented based on the phase of stroke during which MT was applied. The temporal phases of stroke are presented in Figure 1 and are categorized as follows: hyperacute phase (0–24 h); acute phase (1–7 days); subacute phase divided into early (7 days–3 months) and late (3 months–6 months) subacute phase; and chronic phase (>6 months) [18].

## 2. Mirror Therapy in the Hyperacute and Acute Phase of Stroke

To our knowledge, no MT trial has been conducted during the hyperacute and acute phase of stroke.

## 3. Mirror Therapy in the Subacute Phase of Stroke

Several researchers have studied the application of MT during the subacute phase of stroke. Some studies have been conducted exclusively during the early subacute phase (7 days–3 months), while others have addressed the application of MT during both early and late subacute phases. No study was found to have examined the application of MT merely in the late subacute phase (3–6 months). Notwithstanding the phase, the reported results have been rather conflicting.

### 3.1. Early Subacute Phase

Invernizzi et al. evaluated the role of MT in 26 patients with ischemic subacute stroke (<4 weeks) who presented with moderate to severe upper limb hemiparesis (Motricity Index ≤ 77). Patients were randomly assigned to two groups. The MT group received five sessions of MT per week (30 min for the first 2 weeks and 1 h for the following 2 weeks) in addition to conventional therapy, whereas the control group received sham therapy (mirror covered with paper) of the same design and duration along with conventional treatment. The conventional rehabilitation program included 1 h sessions of common neurorehabilitation techniques, combined with occupational therapy and electrical stimulation. MT involved observation of the movements of the unaffected upper limb, which included flexion and extension of the shoulder, elbow and wrist, as well as pronation and supination of the forearm. The patients were assessed at baseline and at the end of the 4-week treatment with the use of the action research arm test (ARAT), the motricity index for the upper extremity (MI-UE) and the functional independence measure (FIM) [10]. ARAT measures the function of the upper limb by rating (on a four-point scale) the patient’s ability to perform 19 functional tasks categorized in four subscales (grasp, grip, pinch and gross movement) [19]. MI-UE assesses motor impairment by grading (on a 0–100 scale) the strength of the paretic upper limb during shoulder abduction, elbow flexion and pinch grip [20,21]. FIM evaluates the functional status of patients by assessing their degree of disability or dependence on 18 activities of daily living, 13 of which involve motor functions (self-care, sphincter control, transfers, locomotion) and 5 of which involve cognitive functions (communication, social cognition) [22]. Although both groups demonstrated improvement in all outcome measures after 1 month of treatment, the improvement was significantly greater in patients who received MT, not only in terms of motor recovery but also in the domain of independence in performing activities of daily living [10].

In a single-blinded randomized trial, 36 patients with severe hemiparesis due to ischemic stroke in the territory of the middle cerebral artery (<8 weeks) were allocated to either an MT group or a control group in which no mirror was present, thus allowing a direct view of the paretic upper limb. Patients in both groups were additionally instructed to perform active movements of the paretic arm as much as possible, while they also received conventional therapy which consisted of physiotherapy, occupational therapy and training in activities of daily living. The main outcome measure was the improvement in the Fugl-Meyer assessment test for the upper extremity (FMA-UE) [11], which assesses sensorimotor function and includes motor subscales for the proximal arm (shoulder, elbow, forearm), wrist, and hand/fingers [23]. The patients were also assessed before and after the intervention by means of ARAT and FIM [11]. Interestingly, although the observed improvement in motor function based on FMA-UE did not differ significantly between the two groups, the MT group showed a tendency of greater improvement in the motor subscale of hand/fingers, which, however, did not reach statistical significance and was found to be driven by a significant difference in the subgroup of patients who were distally hemiplegic. Indeed, when further examining the subgroup of 25 patients who initially had complete hemiplegia, it was revealed that MT resulted in a significantly more pronounced improvement in the distal motor function of the hand/fingers compared to the control group. Among this patient subgroup, it was observed that more patients in the MT group demonstrated functional improvements in the ARAT scale in comparison to the control group. No differences were evident in the FIM scale between the two groups. The authors concluded that MT exerts more prominent effects on the recovery of distal motor function (hand/fingers) [11].

Likewise, in another small-scale study of 15 patients with ischemic subacute stroke (1–3 months), MT was found to result in significant motor improvements of the distal upper limb, compared to conventional therapy, especially with regard to finger flexion based on the Bhakta test (finger flexion scale), as well as the spasticity of the wrist based on the Ashworth scale. The MT group also showed enhanced motor recovery after 6 weeks of intervention, when assessed with the use of the Brunnstrom stages of stroke recovery (BSSR) [24], which is a motor measure classifying motor function into six stages based on the degree of spasticity, synergies and voluntary movement [25]. A similar improvement in the MT group was seen when using FMA-UE [24]. Furthermore, patients in the MT group displayed improvements in the flexion, extension, pronation and supination of the wrist, as well as flexion of the elbow, whereas patients in the control group experienced improvement only in wrist pronation. Neither group showed any statistically significant improvement in shoulder movements [24].

On the contrary, other studies have found that the application of MT in the early subacute phase did not provide any additional benefits on the recovery of the upper limb’s motor function. Yeldan et al. were one of the few research groups who assessed the effect of MT in the very early subacute phase of stroke (mean time since stroke 9.3 days). This pilot study included eight inpatients with ischemic stroke who were allocated to two groups receiving either MT and neurodevelopmental treatment or only neurodevelopmental treatment for 3 weeks. Motor recovery was assessed with FMA-UE and MI-UE, as well as with the stroke upper limb capacity scale (SULCS) [26], which is actually a stroke-specific measure of the upper limb’s basic and advanced capacity to execute 10 functional tasks [27]. The patients were also assessed for their level of functional independence by means of the Barthel index (BI) [26], which measures the patient’s ability to perform 10 activities of daily living related to self-care and mobility without assistance (higher scores indicative of greater degree of independence) [28]. Regardless of the assessment tool used, MT was found to confer no additional benefit with regard to the improvement of the upper limb’s motor function [26].

Similar results were reported in another single-blind randomized controlled trial, which included 35 patients with severe paresis of the upper extremity (Motricity Index < 57) due to ischemic stroke in the very early subacute phase (mean time since stroke 13.3 days). The MT group performed bilateral movements for 30 min twice a day for 4 weeks, whereas the control group performed the same exercises with both arms, but without an interfering mirror. Both groups received additional conventional treatment, which consisted of physiotherapy, occupational therapy, and speech therapy/psychological support (if needed). The upper limb’s motor function was assessed by FMA-UE and the Wolf motor function test (WMFT) [29], which is a 17-item performance measure that assesses the motor ability of the upper limb while performing functional tasks in terms of completion time, functional dexterity and strength [30]. At the end of the intervention, both groups exhibited similar motor recovery in both assessment tools [29].

Likewise, Antoniotti et al. conducted a randomized controlled trial in 40 patients with early subacute stroke (<4 weeks). The patients were randomized to receive either MT with movements of the unaffected arm only, or sham therapy of the same design in which patients were looking at the opaque (instead of the reflective) surface of the mirror. Both groups were also engaged in a conventional rehabilitation program, which included physiotherapy and occupational therapy. FMA-UE, ARAT and FIM were the assessment tools used. Although both groups showed significant improvements in all outcome measures after 6 weeks of intervention, no statistically significant difference was present in the motor recovery of the upper limb between MT and sham therapy [31].

Compared to conventional therapy, MT was found to exert no additional effect on the motor function of the patients’ paretic arm and hand in a Polish study, which included 60 right-handed patients with hemiparesis after a subacute (8–10 weeks) ischemic stroke. In this study, patients were equally randomized to an MT group which received MT along with conventional treatment, and a control group which underwent only conventional therapy. Each group was further divided into two subgroups, based on whether patients suffered from hemiparesis of the right or the left upper limb. The upper limb’s motor function was assessed with the use of two tools [32]: the Frenchay arm test (FAT), which assesses the upper extremity’s motor function by scoring the patient’s ability to perform certain functional tasks [33], and the motor status scale (MSS), which evaluates and grades isolated and complex movements of the shoulder, elbow/forearm, wrist and hand/fingers [34]. Moreover, patients were assessed for their independence in activities of daily living by means of the Functional Index ‘’Repty’’ (FIR), which is a Polish modification of the FIM scale, consisting of 15 instead of 18 items related to activities of daily living [35]. Although both groups had better motor function after 21 days of intervention, the MT group did not show any statistically significant improvement compared to the control group, regardless of the paretic side. The only significant improvement in favour of the MT group over the control group was observed in the domain of independence in self-care activities, but only in the subgroup of patients with right arm paresis who received MT [32].

Finally, a randomized controlled study examined the role of MT, when applied either as an individual or as a group intervention. In total, 60 patients with severe paresis of the upper extremity due to subacute stroke (<3 months) were included in the study. Patients were allocated into 3 groups. The first group received MT on an individual basis, the second group received MT in the form of group therapy in which the therapist treated between two and six patients at the same time, and the third group was the control group undergoing sham therapy in which a wooden board was used instead of a mirror. Assessment tools included FMA-UE, ARAT, BI and the modified Ashworth scale (MAS) [36], which measures spasticity by assessing an increase in muscle tone on a six-point scale with higher scores indicating increased resistance to passive movement [37]. Regardless of the tool used, neither individual MT nor group MT were found to be more effective than control therapy in terms of improving motor function, spasticity or activities of daily living [36].

The basic features of the studies performed during the early subacute phase that led to either positive or neutral results are summarized in Table 1 and Table 2, respectively.

### 3.2. Late Subacute Phase

No MT trial was found to have been conducted exclusively in the late subacute phase of stroke (3–6 months).

### 3.3. Both Early and Late Subacute Phases

With regard to the subacute stage of stroke, most studies have been designed to recruit patients within the whole spectrum of the subacute stage (<6 months), thereby including both the early and late subacute phases.

Gurbuz et al. investigated the effect of MT in 31 hemiparetic patients who had suffered a stroke within the past 6 months (mean duration since stroke 44.3 days). The patients were randomized to receive either MT consisting of simple movements of the unaffected upper limb, or sham therapy in which patients were performing the same movements against the opaque surface of the mirror. At the end of the 4-week intervention, both groups displayed improvements in the motor function of the paretic limb based on FMA-UE and BSSR, as well as in the domain of self-care independence according to FIM. However, in the comparison between the two groups, only the improvement in the FMA-UE score was found to be statistically more significant in favour of the MT group. The authors commented that the presence of a statistically significant difference between the two groups only in FMA-UE, but not in BSSR, might be due to the fact that the patients in the MT group were performing movements with the distal (wrist and fingers) and not the proximal part of the upper limb [38]. Identical results were reported in another study of a similar design, which included 60 patients with motor deficits of the upper arm due to stroke within the last 6 months (mean duration 51.6 days). The only difference from the aforementioned study design was that patients were performing functional tasks with both arms, instead of simple movements with one arm. After 4 weeks of treatment, patients in the task-oriented MT group experienced greater motor recovery according to FMA-UE (but not BSSR), compared to the sham therapy group. Moreover, they demonstrated higher independence in activities of daily living, as assessed by the modified Barthel index (MBI) [39].

Likewise, significantly greater improvement in FMA-UE, but not in BSSR, was observed with the application of task-based MT compared to occupational therapy in a randomized controlled trial of 30 patients with moderate to severe hemiparesis of the upper limb due to subacute stroke (<6 months). Moreover, the group of patients who received MT displayed improvement in spasticity according to MAS, but only for certain movements, which included elbow flexion, wrist flexion and extension, and finger extension (excluding the thumb). When independence in activities of daily living was evaluated, both groups showed improvements in the MBI scores with the application of MT and occupational therapy, respectively, albeit with no statistically significant difference between them [40].

Contrary to the three previous trials, another randomized controlled study found that MT resulted in significant improvements in both FMA-UE and BSSR [12]. In this study, patients received MT with bilateral arm training and performance of graded activities, on top of conventional treatment, and were compared with a group of patients receiving only conventional therapy. The patients in the MT group also achieved greater degrees of hand dexterity, when assessed by the box and block test (BBT) [12], which is a performance-oriented assessment tool of gross manual dexterity that measures the patient’s ability to transfer cubes from one compartment of a box to another within 1 min [41]. With regard to spasticity assessed by MAS, it was not found to be affected by MT. The authors argued that the improvement in motor control observed in both proximal and distal parts of the paretic upper limb might be related to the fact that MT was combined with bilateral arm training [12]. Similarly, Lee et al. reported significantly greater motor improvements in both FMA-UE and BSSR when MT was administered to patients with subacute stroke, on top of conventional therapy. Enhanced motor recovery was evident in both the distal and proximal components of FMA-UE and BSSR. The only subscale of FMA-UE in which improvement was not statistically significant between the MT and the control group was the coordination subscale, most probably because the main focus of the MT intervention was movement induction rather than movement accuracy or speed [42]. Moreover, in the MT group, significant improvement was also observed in all domains of the Manual Function Test (MFT) [42], which assesses the upper limb’s motor function in eight tasks, four of which are related to proximal arm movements and four to manipulation tasks requiring fine and gross dexterity of the hand [43].

Along the same lines, MT was found to exert beneficial effects on the motor function of the paretic upper extremity, which was assessed by means of MFT, in a study of 20 patients with stroke within the past 6 months. The patients were assigned to either an MT group in which patients were performing bilateral movements of the upper extremities or a control group in which the patients were receiving sham therapy (without the presence of a mirror) and were performing unilateral movements only with their paretic upper limb. Although both groups showed motor improvements in MFT, the MT group improved to a greater magnitude than the control group after 4 weeks of intervention. In this study, the researchers also investigated brain activity during the intervention by measuring brain waves at the primary and supplementary motor areas, focusing on mu rhythm suppression, which reflects activation of the corresponding brain areas. By doing so, they observed that MT led to significantly higher activation of the cortical motor areas compared to control therapy, which in turn resulted in enhanced motor function of the paretic upper limb, as assessed by MFT [44].

Positive effects of MT were also reported in a more recent study, which randomized 52 right-handed patients with subacute stroke (<6 months) into 2 groups. One group received MT in addition to conventional therapy, while the other received only conventional therapy. The primary outcome measure was FMA-UE, in which the MT group achieved significantly higher scores than the control group after 3 weeks of treatment. MT also resulted in a significantly increased level of functional independence [45], as assessed by Lawton’s instrumental activities of daily living scale (LIADL) [46]. However, no significant difference was evident in the ARAT tool between the two groups. In a subgroup analysis, it was demonstrated that the efficacy of MT was not affected by age, type of stroke, lesion side or time period since stroke [45]. Likewise, in a similarly designed study, 10 sessions of MT on top of standard therapy were found to be more effective than conventional treatment in enhancing both motor recovery (as assessed by BSSR) and level of independence in self-care activities (as assessed by FIM) [47].

In the study of Cacchio et al., the application of MT was shown to improve the motor function of the paretic upper limb to a greater extent than control therapy over a 6-month period of follow-up. Motor function was assessed by using WMFT and Motor Activity Log (MAL), a disability scale which measures both quantitatively and qualitatively the ability of patients to use their paretic upper limb in 30 activities of daily living. The study included 48 patients with subacute stroke (<6 months) who were also suffering from complex regional pain syndrome type 1 due to stroke. The patients were allocated either to an MT group or to a control therapy group receiving sham therapy with the reflective surface of the mirror covered with paper. The primary end-point of the study was the reduction in pain both at rest and on the movement of the affected upper extremity, as well as the improvement of tactile allodynia. Motor improvements in WMFT and MAL were secondary outcome measures. The patients were assessed at the end of the 4-week intervention and at 6 months from baseline. All outcome measures were found to be significantly more improved in the MT group compared to the control group, not only at the end of the intervention but also at the 6-month follow-up [48]. Nevertheless, since the study included patients with complex regional pain syndrome type 1 after stroke, it cannot be ruled out that the observed motor improvements could be merely due to the effect of pain reduction (which may have abolished the pain-induced restriction of movements) and not to pure motor recovery of the paretic upper limb.

In contradiction to the aforementioned trials, a small-scale study of 12 patients with subacute stroke found that MT had no additional beneficial effect on the recovery of the paretic hand’s motor function when compared to a task-oriented technique based on the principles of the motor relearning program. The patients were evaluated with the use of the Chedoke arm and hand activity inventory (CAHAI) [49], which is a performance measure of the patient’s ability to complete 13 functional tasks that require bimanual involvement [50]. After a 4-week intervention, the two therapeutic approaches did not differ significantly in terms of improving the function of the paretic hand [49].

Table 3 summarizes MT studies in which the application of MT involved both the early and late subacute phases of stroke.

## 4. Mirror Therapy in the Chronic Phase of Stroke

A considerable number of studies have investigated the effect of MT when applied during the chronic phase of stroke (>6 months).

A study of particular interest was conducted by Michielsen et al. in a cohort of 40 patients with stroke (mean time since stroke 3.9 years), who were randomly assigned to receive either MT or sham therapy (same bimanual exercises with no mirror present) for 6 weeks. Both groups received unsupervised home-based treatment on a daily basis (5 times a week), while therapy was also delivered at a rehabilitation center once a week under the supervision of a physiotherapist. Motor function was measured by means of FMA-UE, which was the primary outcome. The patients were also assessed for their grip strength (with a Jamar handheld dynamometer), spasticity (with the use of the Tardieu scale), motor dexterity (with the ARAT), self-perceived and actual performance while using their hands in activities of daily living (with the ABILHAND questionnaire and with the stroke upper limb activity monitor, respectively), quality of life (with the EQ-5D), and level of pain (with a visual analogue scale). Moreover, in a subgroup of 16 patients (9 in the mirror group and 7 in the control group), functional magnetic resonance imaging (fMRI) was performed at baseline and after treatment in order to investigate potential changes in the activation pattern of neural circuits. Upon completion of treatment (at 6 weeks), there was a statistically significant improvement in FMA-UE in the mirror group compared to the control group, which however lost its statistical significance at 6-month follow-up. No changes were recorded in the other secondary outcome measures. Regarding fMRI results, an altered pattern of cortical activation in the primary motor area was observed only in the mirror group, as evidenced by an interhemispheric shift of the activation sequence towards the affected hemisphere, consistent with the induction of cortical reorganization after applying MT [51].

Positive effects of MT were also reported in another trial which included 30 patients with chronic stroke (>6 months) who received either MT or sham therapy for 4 weeks and were assessed post-treatment with the use of FMA-UE, BBT and FIM. Compared to the control group, patients in the MT group experienced significantly greater improvement in the motor function of the paretic upper limb, in the manual dexterity of the affected hand and in the domain of self-care [52]. In keeping with the previous trial, another study of 25 post-stroke patients from South Korea concluded that task-oriented MT resulted in better motor function (as assessed by FMA-UE), greater ability to perform functional tasks (as assessed by ARAT and BBT) and a higher level of independence in activities of daily living (as assessed by FIM), when compared to task-based conventional therapy [53]. Similar results in favour of MT were also reported in another Asian study of patients with chronic stroke, originating from Malaysia [54].

In a single-blinded, randomized controlled study, 33 outpatients with mild to moderate motor deficits in the chronic phase of stroke were assigned either to an MT group or to a control group. The MT group received 60 min of MT followed by 30 min of task-based conventional therapy for 4 weeks, whereas the control group received 90 min of task-oriented conventional therapy. In comparison to conventional treatment, MT had a significantly greater effect on the motor function of the distal, but not the proximal, part of the upper extremity, as evidenced by higher FMA-UE scores of the wrist and hand, but not of the shoulder, elbow and forearm. Moreover, MT led to the improvement of certain kinematic parameters, namely reaction time, normalized total displacement and maximum cross-correlation between the shoulder and the elbow. These improvements translate into better motor preplanning, straighter movement of the upper extremity and better coordination between the joints of the shoulder and elbow, respectively. In other words, MT enhanced motor control of the paretic upper limb by producing more normalized movements. As far as activities of daily living were concerned, patients in the MT group did not experience any significant improvements either post-treatment nor at 6-month follow-up, when assessed by the MAL and the ABILHAND questionnaire [55].

By the same token, a small-scale pilot study of 13 patients with chronic stroke found that the application of task-based MT led to significant improvements in the post-treatment versus pre-treatment scores of FMA-UE only for the distal part of the paretic upper limb, but not for the proximal one [56]. A few years later, the same investigators corroborated the preliminary findings of their previous single-group design trial by conducting a randomized controlled study in 33 post-stroke patients. This time, the patients were randomized to two groups: an MT group receiving 45 min task-based MT followed by 45 min standard occupational therapy, and a control group receiving 90 min conventional occupational therapy for a total of 8 weeks. After 8 weeks, MT mainly enhanced motor recovery of the distal part of the paretic limb (wrist and hand). Interestingly, the MT group experienced significantly greater improvement not only in the FMA-UE subscore for the wrist and hand but also in the total score of the FMA-UE for the entire upper limb, which was, however, most likely driven by the improvement in the FMA-UE subscore for the distal extremity. Apart from that, some patients in the MT group exhibited improvement in the BSSR stage, whereas no patient in the control group advanced to a higher BSSR stage [57].

In a similar fashion, improvement in the motor function of the distal part of the paretic upper extremity was also observed in a single-blinded, randomized controlled trial from Hong Kong, which included 101 patients with chronic stroke. The patients were randomized to receive either 12 sessions of 30 min MT with bilateral arm movements (2 sessions per week for 6 weeks) or bilateral arm training of the same duration with no intervening mirror. At the end of the 6-week intervention, patients in both groups were assessed by means of FMA-UE, WMFT and ARAT. Both groups exhibited improvements in all assessment tools, except for the gross movement subscale of ARAT. However, MT was found to be more effective in improving distal motor function of the paretic limb, since it resulted in significantly greater improvement in the hand subscore of FMA-UE compared to bilateral arm training [58]. Likewise, in a study of 14 patients with chronic stroke, it was found that 4 weeks of MT led to statistically significant improvement in the MFT items which included manipulation tasks related to fine and gross dexterity of the paretic hand, but not in the MFT items related to proximal arm movement [59].

MT was also shown to exert beneficial effects on the motor functions of the paretic hand in patients with ischemic stroke in the chronic phase. In this Egyptian study, patients were assessed in the following fields: change in range of motion of wrist extensors and forearm supinator by using an electronic goniometer; change in hand grip strength with the use of a handheld Jamar dynamometer; and change in motor functional skills of the hand with the use of the Jebsen–Taylor hand function test (JTHFT) [60]. JTHFT is a performance assessment tool which evaluates hand motor function in seven tasks commonly used in activities of daily living, by measuring the time needed to complete each task (faster times indicate better performance) [21]. After 8 weeks of treatment, patients in the MT group demonstrated statistically greater improvement in all assessment tools of hand motor function compared to the control group [60].

Furthermore, in a randomized controlled trial from Italy, which included 24 patients with chronic stroke who also suffered from chronic complex regional pain syndrome type 1, it was found that MT was more effective than sham mirror therapy (covered mirror) or mental imagery therapy, in terms of improving motor function of the paretic limb, as assessed by WMFT [61]. Likewise, in patients with chronic stroke and shoulder-hand syndrome, it was shown that MT enhanced activities of daily living (as assessed by FIM) to a significantly greater extent than sham therapy after 4 weeks of treatment. Notably, at 2-week follow-up, patients who had received MT maintained this improvement in functional activities and continued to improve. Considering the fact that this study did not use any specific assessment tool of motor function, the observed improvement was attributed to the reduction of oedema and pain, which were actually the main outcome measures of the study [62].

Paik et al. compared the effects of two different types of MT (simple versus task-oriented MT) on the recovery of the upper limb’s motor function in 4 patients with chronic stroke. During simple MT, patients were performing simple movements (forearm pronation and supination, flexion and extension of the wrist and fingers, finger numbering and opposition), whereas task-oriented MT included the performance of functional tasks related to activities of daily living. Although both simple and task-oriented MT led to improvement of motor function of the paretic upper limb, it was observed that task-oriented MT was more effective in promoting motor recovery. In fact, the treatment effect started to decline after treatment cessation in patients receiving simple MT, whereas improvement was maintained in patients receiving task-oriented MT who actually continued to improve even after the completion of MT [63]. In another study of patients with chronic stroke (>1 year), 12 sessions of MT were found to be more effective than conventional physiotherapy in enhancing the motor function of the paretic upper limb, when assessed by means of WMFT and JTHFT [64].

On the other hand, Colomer et al. reported that MT did not produce any statistically significant beneficial effect when compared to control therapy. In their study, 31 patients with chronic stroke (>6 months) and severe paresis of their upper limb were randomized to either MT or therapy with passive mobilization of their paretic limb for 8 weeks. Both at baseline and after completion of therapy, motor function of the paretic upper extremity was assessed with the use of WMFT and FMA-UE. It was observed that both groups showed improvement in WMFT, but not in FMA-UE. Yet, the treatment effect provided by MT was found to be similar to that provided by passive mobilization [65].

In another randomized controlled trial, 16 patients with moderate motor impairment due to chronic stroke were assigned to receive either MT with object-related bilateral symmetrical training or sham therapy which consisted of the same tasks involving bilateral manipulation of objects, but with a covered mirror. The patients were assessed by means of the FMA-UE tool and the upper extremity performance test for the elderly/Test d’évaluation des membres supérieurs des personnes âgées (TEMPA) [66]. TEMPA is a tool which assesses activity limitation of the upper extremity and includes eight standardized tasks of daily activities (four unilateral and four bilateral) that are scored based on speed of execution, degree of functionality or else level of assistance required for each task, and task analysis (strength, range of motion, accuracy of gross movements, grasp, and accuracy of fine movements) [67]. With regard to both TEMPA and FMA-UE, no statistically significant difference was observed between the two groups after treatment. The authors concluded that MT was not superior to sham therapy in terms of improving motor function and activity level of the paretic upper limb [66].

Neutral effects of MT on motor recovery and motor function of the paretic upper limb were also reported in a study of 23 post-stroke patients with mild to moderate motor impairment who were randomized to a task-oriented MT group or a sham therapy group. The MT group was receiving both hospital-based MT (3 days per week) and home-based MT (5 days per week) for 4 weeks, whereas the sham therapy group was performing the same bilateral tasks without a mirror. Upon completion of treatment, the two groups did not exhibit any significant differences in motor recovery and function, when assessed by FMA-UE and CAHAI. Nonetheless, MT seemed to have a better effect on the patients’ quality of life (as assessed by the Stroke Impact Scale) and the self-perceived performance of their paretic limb during activities of daily living (as assessed by MAL) [68].

Along the same lines, in a small study of six patients with chronic stroke (mean duration of stroke since onset 5 years) and mild to moderate motor impairment, MT failed to produce any significant improvements in either motor function (assessed by means of FMA-UE) or functional independence (assessed with the use of FIM). The patients were allocated to two groups receiving either task-oriented MT or MT based on simple bilateral motor patterns. Post-treatment values of FMA-UE and FIM did not differ significantly compared to pre-treatment values in each group, implying that MT had no significant effect in either group. There were also no significant post-treatment differences during the comparison between the two groups. When both groups were analyzed together as a whole, the only significant difference recorded between baseline and post-treatment values was in the cognitive functions of the FIM, albeit not in the motor ones [69].

When 32 patients with chronic stroke were randomized to receive either MT combined with isometric unilateral exercises (MT group) or cross-education therapy based on isometric strength training exercises of their unaffected upper limb (control group), it was found that MT failed to outperform control therapy in terms of improving motor function, spasticity, functional ability in activities of daily living or quality of life [70]. Likewise, in a study comparing different training schemes with the use of a mirror or not and with the use of unimanual or bimanual movements, MT was not found to be superior to direct training of the paretic upper limb only, in terms of improving movement time needed to execute a certain task [71].

The aforementioned studies conducted in the chronic phase of stroke are summarized in Table 4 and Table 5, based on whether the studies showed positive or neutral effects of MT on motor recovery, respectively.

## 5. Mirror Therapy in More than One Phase of Stroke

Several studies of MT have been conducted in more than one phase of stroke, spanning from acute to subacute or from subacute to chronic phase, while other trials have not specifically defined the phase of stroke during which MT was applied.

The only study to have been conducted during the acute and early subacute phases of stroke was a pilot study from India, which included 11 patients with the duration of stroke ranging from 2 to 45 days. The patients received 50 min of MT in addition to conventional rehabilitation and were assessed by using the hand subsections of the FMA-UE and WMFT tools, both at baseline and upon completion of treatment. After 4 weeks of therapy, it was reported that MT resulted in significant improvements in the motor function of the paretic wrist and hand with both assessment tools [72].

Yavuzer et al. conducted a randomized controlled trial, which included 36 patients who had suffered a stroke within the previous 12 months and were allocated to an MT group or a sham group. The patients were assessed after 4 weeks of therapy and at 6-month follow-up by means of BSSR, MAS and FIM. At both time points, MT was found to enhance motor recovery of the paretic upper limb and self-care functioning to a significantly greater extent compared to sham therapy. However, it did not appear to affect spasticity [73]. Likewise, in a cohort of 35 patients with hemiparesis after stroke (duration <1 year), MT was found to be more effective than sham therapy after 4 weeks of treatment, in terms of promoting motor recovery (assessed by BSSR) and improving functional performance (based on ARAT) and level of independence in self-care (based on FIM). Yet, MT failed to improve spasticity (based on MAS). Interestingly, at 6-month follow-up, significant improvement was maintained only in the self-care domain, whereas improvement in the BSSR and ARAT parameters was no longer statistically significant [74]. Positive effects of MT on both the paretic upper limb’s motor function and ability to perform activities of daily living were also reported in a study of 30 patients who had a stroke within the previous 2–12 months [75].

The effect of MT was also tested in a group of 30 patients who had suffered a stroke within the last 12 months, complicated by complex regional pain syndrome type 1. The patients were divided into two groups: an MT group receiving 30 min of MT in addition to a conventional rehabilitation program and a control group receiving only conventional therapy. The assessment tools used for evaluating changes in motor impairment included BSSR, MAS, the wrist and hand subsections of FMA-UE, and the motor items of FIM. After 4 weeks of treatment, MT resulted in significant improvements in BSSR, FMA-UE and FIM, whereas it did not have any significant effect on spasticity. When compared to the control group, the MT group experienced significantly greater improvements in FMA-UE and FIM [76].

A randomized controlled study from Thailand followed a different design in order to investigate the effect of adding MT to conventional rehabilitation programs in 40 patients with stroke (time since onset > 3 months). MT was delivered for 2 weeks, whereas the conventional rehabilitation program was applied for 8 weeks. Patients were evaluated at baseline, at 2 weeks (upon completion of MT), at 4 weeks, at 8 weeks (upon completion of conventional therapy) and at 12 weeks. The assessment tools used were BSSR, MAS, the Barthel index (BI), the motor assessment scale of the upper extremity, and the pinch gauge for measuring tip and lateral pinch strength. The patients were randomized to receive either 2-week MT or 2-week sham therapy (using the non-reflecting surface of the mirror) on top of conventional therapy. Patients in the MT group demonstrated significant improvements in the BSSR scales for the arm and the hand, and in BI, as early as 2 weeks, which were maintained at 12 weeks. Moreover, in the MT group, the motor assessment scale improved at 8 weeks and continued to improve at 12 weeks, whereas no significant improvement was observed in MAS and pinch strength. The sham group showed improvement only in the BSSR scale for the arm (but not the hand) and in BI at 2 weeks, which continued throughout the 12-week follow-up. When comparing the two groups, only the improvement in the BSSR scale for the hand was found to be statistically more significant in favour of the MT group at 2 weeks, which, however, lost its significance beyond that time point [77].

Interestingly, when MT was compared to task-based training of the paretic upper limb, it was found to be inferior to task-based training, whereas the combination of MT and task-based training produced the best results. In this comparative study, 37 post-stroke patients were randomized to three groups: the first group received only MT with movement of the unaffected upper limb; the second group received only task-based training of the paretic upper extremity; and the third group received both MT and task-based training. After 4 weeks of treatment, it was found that all groups demonstrated significant improvements in motor recovery and function, as assessed by FMA-UE and ARAT. However, it was noted that the group receiving both therapies exhibited the greatest improvement, followed by the group undergoing task-based training, whereas the group receiving only MT showed the smallest improvement [78].

Likewise, in a cohort of 66 post-stroke patients, MT was found to be less effective than a motor relearning programme in enhancing the motor function of the paretic upper limb. The patients were randomly assigned to receive either MT or therapy based on a motor relearning programme consisting of different types of task-oriented exercises with varying levels of difficulty. After 6 weeks of therapy, the patients in the motor relearning programme group exhibited greater improvements in the upper limb subscales of the motor assessment scale (upper limb function, hand movements and advanced hand activities) compared to the patients receiving MT [79].

Finally, a Chinese study, which included 60 patients with stroke and post-stroke depression, reported that MT outperformed occupational therapy in terms of improving motor function and independence in activities of daily living. The patients were randomized to receive either occupational therapy or MT on top of occupational therapy for 4 weeks. Upon treatment completion, the patients were assessed by means of FMA-UE and MBI. The MT group experienced greater improvements in both assessment tools, in comparison to the occupational therapy group. Furthermore, MT was found to be more effective than occupational therapy in alleviating concomitant depressive symptoms [80].

Table 6 summarizes MT studies which were conducted in more than one phase of stroke.

## 6. Discussion

MT, originally termed virtual reality box, was first introduced by Ramachandran et al. as a method for alleviating the pain of a phantom limb in a small cohort of upper limb amputees [81,82]. Subsequently, in 1999, Altschuler et al. investigated the application of MT in patients who had previously suffered a stroke, with the aim to facilitate the recovery of their paretic upper limb [83]. Driven by the beneficial effects on the paretic arm’s motor function reported in this initial trial, many studies of MT have been conducted in patients with stroke ever since [84,85,86], while at the same time, the use of MT has been further extended to incorporate a variety of conditions, including cerebral palsy [87], chronic pain syndromes [88], Parkinson’s disease [89], fractures [90], and hand injuries [91].

Although the exact neurophysiological mechanisms underlying MT have not been fully clarified and are still vague, several theories have been put forward. By exploiting visual feedback through the use of a mirror, MT is thought to activate the mirror neuron system, which represents a motor-related cortical network responsible for learning new motor skills through observation of goal-directed motor actions. The mirror neuron system is widely distributed in several areas of the cerebral cortex, residing mainly in the parietal lobe (intraparietal sulcus and its adjacent superior and inferior parietal lobule), the frontal lobe (premotor cortex and inferior frontal gyrus) and the superior temporal sulcus, and consists of a circuit of bimodal (visual and motor) mirror neurons which discharge both when observing a given motor action and when executing the same action [92,93,94]. Apart from the classic cortical circuitry, it has been recently demonstrated through functional neuroimaging studies that the mirror neuron system also extends to subcortical areas, including the basal ganglia, the thalamus and the cerebellum, which seem to be activated during action observation, especially when the action involves gripping and grasping tasks or goal-directed manipulative tasks. In particular, the cerebellum seems to be involved in observation-based motor learning through perceiving and monitoring the sequential kinematics and temporal dynamics of the observed action. By doing so, the cerebellum reinforces the establishment of sensory-motor patterns and results in visuomotor matching by modulating the activity of cortical inhibitory interneurons with mirror properties. In parallel, the basal ganglia are mainly engaged in inducing or inhibiting the actual execution of the observed action, whereas the thalamus seems to provide general motor and visual information through its sensory-motor and visual nuclei. Collectively, the cerebellum and the subcortical structures act by regulating and fine-tuning the main cortical component of the mirror neuron system via processing specific aspects of both action observation and execution. Taken together, the mirror neuron system is a complex execution–observation matching system consisting of cortico-cerebellar circuits, which are reciprocally interconnected via cerebellar-thalamo-basal ganglia–cortical loops and are thought to be activated during action observation and action execution, as in the case of MT [95,96,97].

It has been proposed that MT acts by reducing the learnt non-use phenomenon (a phenomenon whereby patients with stroke learn to avoid the use of their paretic limb) through an increase in spatial attention towards the paretic limb. To this end, it has been shown that MT activates brain areas related to cognitive processing, alertness, self-awareness and spatial attention, such as the precuneus and the posterior cingulate cortex [98,99]. Furthermore, it has been postulated that MT reduces asymmetrical activation between the two hemispheres and reestablishes a new state of balance by shifting the inter-hemispheric equilibrium across the transcallosal pathway in favour of the lesioned hemisphere. Indeed, MT seems to trigger several neuronal networks and induce brain reorganization and cortical rewiring by promoting neuroplasticity changes in the primary motor cortex and increasing the excitability of the corticospinal pathway [99,100,101,102,103]. When cortical activity was measured with the use of magnetoencephalography in patients with stroke undergoing MT, it was found that MT enhanced the initial asymmetry in movement-related beta desynchronization by establishing a more normalized and symmetrical desynchronization pattern between the primary motor cortices of the two hemispheres [100]. Moreover, cortical reorganization processes, indicative of increased neuroplasticity, have been investigated in patients with subcortical stroke by means of electroencephalography and event-related potentials and it has been demonstrated that event-related desynchronization during action observation could predict motor recovery, since the presence of decreased activity in the unaffected hemisphere, combined with increased lateralization of the activity towards the lesioned hemisphere, was associated with better functional outcomes [104]. Similarly, the use of electroencephalography biomarkers, such as the brain symmetry index (BSI) and the delta alpha ratio (DAR), as well as kinematic parameters reflecting movement duration and movement smoothness, has been shown to predict early motor recovery of the upper limb in patients with stroke [105]. It should be emphasized that kinematic parameters, contrary to clinical subjective parameters, represent an objective measure of motor control by incorporating both spatial and temporal characteristics of upper extremity movements. Such kinematic data may provide solid evidence about the actual effects of MT on motor skills and strength of the upper limb [55].

MT shares many common grounds with action observation therapy, since they are both based on observation of an action followed by execution of the action. However, the patterns of motor observation, imitation and execution differ between these two therapeutic modalities. Indeed, MT further utilizes the phenomenon of optical illusion in order to deceive the brain into a false reality, whereby the reflection of the unaffected upper limb on the mirror creates the illusion that both upper limbs are synchronously moving since the paretic limb is perceived to be moving as if it were the intact limb. On the other hand, action observation therapy entails observation of the actual actions performed by another person, succeeded by the execution of the exact same actions that have been previously observed by the patients themselves [106]. In this regard, MT promotes neuroplasticity by primarily exploiting visual and proprioceptive feedback from the intact upper limb, as a substitute to compensate for the diminished input from the paretic arm, whereas action observation therapy mainly involves motor understanding, learning and execution of actions with equal contribution from both arms [106]. Although both therapeutic approaches are thought to activate the mirror neuron system, the activation pattern is different, given that action observation therapy evokes a much wider activation of frontal, parietal, temporal and occipital regions, whereas MT mainly activates frontal and parietal areas [107]. It is also noteworthy that only MT, but not action observation therapy, induces lateralized cerebral activation by selectively activating the lesioned hemisphere and thus shifting the inter-hemispheric balance away from the unaffected and towards the affected hemisphere [108].

Regardless of the underlying neurophysiological mechanism, MT has been extensively applied in clinical practice, even though there is still a lack of solid indisputable evidence on its effectiveness in patients with stroke. To this end, there has been a wealth of clinical trials investigating the role of MT as a rehabilitation method for patients with motor deficits of the upper limb due to stroke. Related studies have examined the effects of MT in different phases of stroke, spanning from acute to chronic phase. This is rather intriguing, especially when taking the natural history of recovery into consideration. After a stroke, two separate mechanisms come into play: recovery (spontaneous biological recovery and true behavioural restitution) and compensation. Early post-stroke, there is a time-sensitive window lasting approximately 3 months, during which spontaneous recovery occurs in the absence of any treatment, with the recruitment of brain repair processes that are initially intense and gradually taper off. After 3 months, spontaneous recovery wanes off and compensatory mechanisms take over; hence, any improvement seen beyond 3 months would be almost entirely attributed to compensation. After 6 months, patients in the chronic phase of stroke are more likely to have reached a plateau in terms of motor recovery. Moreover, the most critical period during the time-sensitive window, wherein the endogenous neural plasticity reaches its peak and thus the largest amount of motor recovery is achieved, seems to extend from the first week till the first month post-stroke. Presumably, this timeframe should be the target for a given rehabilitation intervention in order to achieve its maximum therapeutic potential. However, it may be difficult to differentiate between true and intrinsic spontaneous recovery and discriminate whether a given intervention has actually resulted in true recovery beyond the one which would otherwise occur spontaneously, even without the intervention [18,109,110,111,112]. It should also be noted that researchers have recognized that, beyond the time-sensitive window, a potential for true (behavioural) recovery still exists even years after stroke, albeit to a much lesser extent [18], owing to the fact that compensation is the prevailing mechanism after 6 months post-stroke. Of course, it should be kept in mind that there is considerable inter-individual variability and diversity in cortical reorganization and compensatory mechanisms among different patients.

Notwithstanding the stroke phase, conflicting results have been reported with regard to the effectiveness of MT in promoting motor recovery and function of the paretic upper limb. It is thus difficult to reach firm and definite conclusions regarding the effectiveness of MT in patients with stroke, especially when taking into consideration that there is a great heterogeneity among MT trials in terms of patient characteristics, type of stroke, severity of stroke, phase of stroke, study design, assessment tools, and MT mode delivery. Indeed, MT trials have used different protocols while applying MT with respect to application time, duration of treatment, intensity of therapy and type of allowed movements (unilateral or bilateral movements, simple or complex movements, movements based on functional tasks). Moreover, different rehabilitation programs and different types of treatment have been used in the control groups. All these different parameters limit the interpretation of the study results and render it difficult to make direct and safe comparisons among studies. Moreover, the majority of studies are small-scale, single-center clinical trials of less rigorous design.

It could be argued that the high heterogeneity of several factors, including age of the participants, gender, underlying comorbidities, cognitive deficits, baseline level of motor impairment or initial degree of spasticity, might have contributed to the fact that some studies yielded positive results of MT, whereas others demonstrated neutral effects of MT. Indeed, the mean age of the patients participating in the included studies varied considerably, ranging from 45 [53] to 71 [10] years old. To this end, many researchers have concluded that increasing age negatively affects functional and motor recovery and that older patients with stroke are less likely to experience favourable functional outcomes compared to younger ones [113,114,115]. Patients included in the studies presented with varying levels of motor impairment at baseline, as assessed by different scales of motor or functional status. For instance, Wu et al. [55] recruited patients with mild to moderate motor impairment (FMA-UE scores from 26 to 56) at baseline, whereas the studies of Chan et al. [29] and Wen et al. [45] included patients with initially moderate to severe motor impairment (Motricity Index < 57/100 and FMA-UE < 40, respectively). On the other hand, Radajewska et al. [32] included only patients with severe motor impairment, as did Thieme et al. [36] who recruited exclusively patients with severe distal hemiparesis based on the Medical Research Council grading (0 or 1 for wrist and finger extensors). One could therefore assume that the existing discrepancies in the initial severity of motor deficits may have led to the observed incongruous results of MT effectiveness among the included studies, especially when considering that the most important predictor of the functional outcome seems to be the initial severity of the upper limb’s motor impairment [116,117].

Likewise, different baseline degrees of spasticity would be expected to have a significant impact on the outcome of MT, given that spasticity itself significantly restricts the range of motion and limits the patient’s ability to move their paretic arm in the best possible manner, thus directly interfering with the fundamental principle of MT, which normally requires the execution of bilateral coordinated movements of both the unaffected and the paretic upper limbs. Moreover, even if spasticity allowed a certain degree of movement of the paretic arm, then the movement would again result in increased pain and discomfort, which in turn might diminish the patient’s motivation and initiative to focus on the therapeutic task and render him reluctant to engage actively in the therapy [118,119]. Furthermore, it has been postulated that visual feedback provided by MT might not be sufficient on its own to improve spasticity [12]. Inevitably, the baseline level of spasticity would be expected to pose a significant challenge to the anticipated functional outcomes of MT [120]. Nevertheless, it needs to be emphasized that MT can still be applied in the presence of spasticity. Even more so, it has been shown by Madhoun et al. that MT led to statistically significant improvements in spasticity compared to conventional therapy, which was evident in certain muscle movements corresponding to elbow flexion, wrist flexion, wrist extension and finger extension [40]. This implies that MT has the potential to modify post-stroke pathological synergies, which may develop as a result of the lesioned corticospinal tract and lead to abnormal and fixed movement patterns. In this case, MT probably acts by reorganizing neural circuits, circumventing disrupted neural pathways and reconstructing alternative motor tracks. Through induction of neuroplasticity and motor relearning, MT results in dynamic motor remapping and thus promotes more functional, independent, coordinated and efficient movement patterns [121,122,123].

An interesting finding of the included studies is that improvements in motor function were not invariably accompanied by improvements in activities of daily living. The discrepancy between motor recovery and improvement in daily autonomy functions could be attributed to the fact that measures of functional independence usually assess activities which require the use of several parts of the body, thus incorporating whole-body functions, whereas measures of motor function and functional recovery are focused on a specific part of the body, the upper extremity in this case. Therefore, it is not surprising that improvements in a given measure of motor function do not necessarily translate into improvements in activities of daily living and vice versa [112]. Furthermore, it has been observed that, beyond the limits of their formal training sessions, most patients with stroke do not actively engage in activities of daily living during the remainder of the day for several reasons. Oftentimes, they complain about fatigue, which prevents them from undertaking any physical activity or exercise. Additionally, their low self-efficacy, coupled with fear and low self-confidence, may limit their participation in daily activities. When participating in activities of daily living, they usually tend to avoid utilizing their paretic upper limb and use their non-paretic limb instead in order to complete a certain task, thus promoting the learnt non-use phenomenon, which has detrimental effects on the rehabilitation process [124].

Several studies have shown that MT seems to be more effective in promoting motor recovery of the distal than the proximal parts of the upper limb [11,24,55,56,57,58,59,77]. The less pronounced improvement on the proximal parts could be due to the design of the mirror box itself, which significantly limits or even precludes free movements of the shoulder region. Apart from that, the size of the mirror used in the studies might have played a contributing role, since small-sized mirrors may have hindered the visualization of the proximal parts of the upper limb on the mirror [17,56]. Moreover, MT might have entailed exercises that facilitated movements of the distal rather than the proximal parts of the upper extremity [38,56], while the choice of the assessment tool per se might have additionally created a bias towards favouring the distal parts of the arm [38]. Based on the premise that proximal and distal motor functions are characterized by different representation patterns and disparate levels of contribution from each hemisphere (axial and proximal limb muscles receive bilateral innervation, whereas distal limb muscles are primarily subject to the control of the contralateral cerebral hemisphere) [125,126], Dohle et al. have proposed that MT mainly promotes lateralized (and not bihemispheric) mental representation for the movements of the distal arm in the cerebral cortex [11].

Although MT is a rehabilitation technique highly dependent on attention and information processing [127], most studies did not take into consideration possible confounding factors relating to the attention, motivation and cooperation of patients during treatment sessions. Only a few studies have actually controlled for such potential confounders by separately assessing the patients’ vigilance, alertness and ability to engage in a task with sustained attention during each session [11], while others solely mention that the therapeutic intervention was performed in an environment free from distractors, without specifically estimating patients’ attention [65]. However, it needs to be emphasized that the degree of attention during MT sessions should be meticulously measured in future trials, given that it is an important determinant that can affect the effectiveness of MT, especially when considering that oftentimes patients tend to become easily distracted or observe the unaffected upper limb instead of the mirror during the session, thus failing to maintain focus on the mirror [128].

Some studies reported high rates of drop-out [11,26,36]. This highlights the importance of optimal patient selection before initiating treatment since several factors can affect patient compliance and thus treatment effectiveness, such as fatigue, insufficient trunk control, inability to understand the rationale of MT, loss of attention, cognitive impairment, vision problems, neglect, or health insurance issues. Although the cerebellum has been shown to be associated with the mirror neuron system [95], none of the included MT studies have particularly addressed cases of cerebellar stroke, but instead, the vast majority of them have so far recruited patients with either hemispheric stroke (cortical or subcortical) or stroke of undetermined anatomical location. It would thus be worthwhile conducting future trials which will include patients with cerebellar stroke in order to examine the effect of MT in this patient subgroup and gain deeper insight into the underlying mechanisms of the cerebellum-related mirror neuron system.

Common limitations reported in the studies include the small sample size, the absence of follow-up or the lack of long-term follow-up, the lack of comprehensive cognitive assessment, the lack of blinding of participants and therapists due to the interactive nature of MT, and the lack of neuroimaging that would provide evidence for brain reorganization after MT. In addition, the fact that clinical rating scales entail some degree of subjective clinical judgment cannot be overlooked. Differences in the set-up, the phase of stroke and the severity of motor impairment preclude the generalizability and the extrapolation of the results from each study to other patient populations with different characteristics. Publication bias towards favouring studies with positive results cannot be ruled out, especially when taking the small-study effect into consideration, namely the tendency of trials with a small sample size to report beneficial effects of a particular intervention.

The relatively weak methodological quality of MT studies has been realized in an early systematic review dating back to 2011, which also highlighted the fact that MT studies were very heterogeneous in design, sample size, clinical setting, intervention characteristics and outcome measures. Evidence regarding the effectiveness of MT in the recovery of the upper limb’s motor function was deemed to be of moderate quality [127]. A subsequent systematic review by Thieme et al. concluded that MT may enhance motor function and activities of daily living in patients with stroke and that these beneficial effects seem to be maintained at 6-month follow-up. However, the authors pointed out that the observed benefits might have been affected by the type of treatment in the control group, given that MT was found to be significantly more effective in those trials which used sham therapy with a covered mirror (thus precluding any view of the paretic limb), whereas the statistical significance in favour of MT was not that robust in those studies where the control group was receiving sham therapy with no intervening mirror or with transparent plexiglass (thus allowing unrestricted view of the paretic limb) [129]. A more recent Cochrane review by the same researchers, which included a much larger number of studies and participants, corroborated the previous findings and noted that there is evidence of moderate quality that MT improves motor recovery, motor function and activities of daily living, at least when used as a supplementary method in conjunction with routine rehabilitation. However, the effect of MT on motor function did not seem to remain at 6-month follow-up. Additionally, MT was found to be effective for both subacute and chronic phases of stroke. The systematic review also highlighted the presence of methodological shortfalls in the included studies, mainly owing to small sample sizes and lack of proper reporting of details and results [84]. Another systematic review focused on the protocol parameters that may affect the beneficial outcomes of MT and concluded that patients achieved more functional gain while performing unilateral rather than bilateral movements, when MT training did not involve manipulation of objects and when a large-sized mirror was used [17].

Obviously, many gaps in the evidence base for the effectiveness of MT in stroke rehabilitation still exist. It is therefore not surprising that, in the 2016 guidelines for adult stroke rehabilitation and recovery from the American Heart Association (AHA)/American Stroke Association (ASA), MT has not been included among the rehabilitation methods recommended for the improvement of upper extremity activities [130]. Nonetheless, in the recently published guidelines for stroke rehabilitation in adults from the National Institute for Health and Care Excellence (NICE), MT has been added in the 2023 recommendations as an adjunct rehabilitation therapy that should be considered in patients with stroke and muscle weakness of their upper limbs. The NICE clinical guidelines also moved a further step forward by determining the time period, during which MT should be applied, as well as its duration and frequency. The NICE committee recommended that MT should be started within the first 6 months after stroke onset (ideally within 1 month after stroke) and be delivered in the form of 30-min sessions at least five times a week over a period of 4 weeks [131].

Undoubtedly, MT is a simple, inexpensive, patient-centered and non-invasive method of rehabilitation which can be rather easily applied both in the hospital environment with the aid of a therapist and at home after discharge from the hospital. It is flexible and easily accessible to the patient, considering the fact that it is a low-cost rehabilitation technique which does not require expensive equipment and can be practiced independently by the patient and without supervision by a therapist, after adequate training. More importantly, it is safe and does not seem to have any significant side effects [39,45,72]. As such, it is an appealing therapeutic method that could be incorporated into the armamentarium of stroke rehabilitation and be routinely implemented in patients with stroke and motor impairment of the upper limb, either as a stand-alone method or in conjunction with other rehabilitation modalities, with the ultimate goal to achieve the highest level of functioning and participation possible. In order to do so, high-quality evidence for the effectiveness of MT in promoting motor recovery and occupational performance, derived from future large-scale and meticulously designed trials, is warranted [103,132].

Based on the mechanisms of stroke recovery, it could be assumed that MT would be more beneficial when applied as an adjunct method during the early subacute phase of stroke, in order to augment the inherent mechanisms of neural plasticity and promote true behavioural restitution [18]. Younger patients with stroke would be expected to benefit more from the application of MT, especially those who present with less severe motor deficits, based on both clinical judgment and imaging methods [115]. Patients without any cognitive or attention deficits are more likely to achieve greater gains in motor recovery [127,128]. The intensity and frequency of MT should be high enough in order to produce clinically significant effects. According to the St. Gallen protocol, which dates back to 2008, but its development was not based on studies including patients with stroke, it is recommended that patients begin with 5–10 min of MT approximately five to six times per day [14]. Based on a more recent systematic review, it has been proposed that MT should be delivered in sessions of 20 min per day for 5 days per week for a total duration of 4 weeks [15]. Another systematic review has also recommended that MT should have an average duration of 4 weeks, but it has advocated a much more intensive MT program lasting 2.5 to 7.5 h per week [112]. The most recent NICE guidelines recommend that MT should be initiated within the first month after stroke onset and should consist of 30-min daily sessions for 5 days per week, for at least 4 weeks. MT sessions should be initially supervised by a therapist and after an initial period of training, patients can continue with MT unsupervised either at the hospital or at home, provided that their cognition is intact [131]. Nevertheless, no official guidelines currently exist with regard to the application protocol and the eligibility criteria that a patient with a stroke should fulfil in order to gain the maximum benefits from this rehabilitation technique.

## 7. Conclusions

MT seems to be a promising technique for the rehabilitation of the upper limb’s motor deficits. Despite the fact that the results of MT trials have been rather inconsistent thus far due to the substantial heterogeneity of the studies, MT is widely used in clinical practice as an adjunct method for the rehabilitation of patients with stroke. Given that MT is a simple, inexpensive, practical and readily applicable method for the rehabilitation of a wide range of patients with stroke, there is a need for conducting large-scale, well-designed, multicenter MT studies of rigorous methodological quality in order to reach more robust results regarding the effectiveness of MT [133]. It is crucial for the application protocol of MT to be standardized in terms of duration, intensity, frequency, method of administration and details in individual MT program components. Furthermore, it is imperative to determine the criteria for the optimal selection of suitable patients at the most favourable stage of stroke who would benefit the most from the application of MT. Future carefully designed and well-powered studies could pave the way for providing high-quality evidence in support of MT, either as a stand-alone rehabilitation technique or as an adjunct method combined with other therapeutic modalities for the rehabilitation of the upper limb’s motor function in patients with stroke.

## Figures and Tables

**Table 1:** Studies in the early subacute phase of stroke demonstrating beneficial effects of mirror therapy.

Author Country Year	Stroke Duration and Type	Number of Patients	Study Design	Assessment Tools	Major Finding
Invernizzi et al. Italy 2013 [10]	<4 w Mean 23 d Ischemic type	26	MTG (*n* = 13): Additional 30 min MT (unilateral movements) for first 2 w and 1 h MT for next 2 w per session on top of ST CG (*n* = 13): Equivalent sham therapy (covered mirror) + ST with 5 1-h sessions/w for 4 w	ARAT MI-UE FIM	Patients in the MTG showed statistically greater improvement in all outcome measures of motor function and independence.
Dohle et al. Germany 2009 [11]	<8 w Mean 27 d Ischemic type	36	MTG (*n* = 18): 30 min MT (bilateral movements), 5 d/w for 6 w + ST CG (*n* = 18): Equivalent sham therapy (no mirror) + ST	FMA-UE ARAT FIM	MT was beneficial for the recovery of motor function of the distal part of the upper extremity (hand/fingers) in patients who suffered from complete distal hemiplegia.
Mirela Cristina et al. Romania 2015 [24]	1–3 mo Mean 53.3 d Ischemic type	15	MTG (*n* = 7): 30 min MT (bilateral movements), 5 d/w for 6 w + ST CG (*n* = 8): ST with 5 30-min sessions/w for 6 w	Ashworth scale Bhakta test BSSR FMA-UE	Compared to ST, MT resulted in significantly greater motor improvements in the wrist component of the Ashworth scale and the finger flexion of the Bhakta test.

Abbreviations: ARAT = action research arm test; BSSR = Brunnstrom stages of stroke recovery; CG = control group; d = day(s); FIM = functional independence measure; FMA-UE = Fugl–Meyer assessment test for the upper extremity; h = hour; min = minutes; MI-UE = motricity index for the upper extremity; mo = month(s); MT = mirror therapy; MTG = mirror therapy group; ST = standard therapy; w = week(s).

**Table 2:** Studies in the early subacute phase of stroke demonstrating neutral effects of mirror therapy.

Author Country Year	Stroke Duration and Type	Number of Patients	Study Design	Assessment Tools	Major Finding
Yeldan et al. Turkey 2015 [26]	<1 mo Mean 9.3 d Ischemic type	8	MTG (*n* = 4): 20 min MT (unilateral movements), 5 d/w for 3 w + NDT CG (*n* = 4): NDT with 5 40-min sessions/w for 3 w	FMA-UE MI-UE SULCS BI	There was no additional improvement in any of the outcome measures, when applying MT on top of NDT.
Chan et al. China 2018 [29]	<1 mo Mean 13.3 d Both types	35	MTG (*n* = 15): 30-min sessions of MT twice daily (bilateral movements), 5 d/w for 4 w + 150 min ST CG (*n* = 20): Equivalent sham therapy (no mirror) + 150 min ST	FMA-UE WMFT	MTG and CG demonstrated similar extent of improvement in motor function with both assessment tools.
Antoniotti et al. Italy 2019 [31]	<1 mo Mean 22.7 d Both types	40	MTG (*n* = 20): 30 min MT (unilateral movements), 5 d/w for 6 w + 135 min ST CG (*n* = 20): Equivalent sham therapy (opaque surface of the mirror) + 135 min ST	FMA-UE ARAT FIM	No difference found in the motor recovery of the upper arm between the two groups.
Radajewska et al. Poland 2013 [32]	8–10 w Mean 9.25 w Ischemic type	60	MTG (*n* = 30, 15 with L and 15 with R hemiparesis): 15-min sessions of MT twice daily (bilateral movements), 5 d/w for 21 d + ST CG (*n* = 30, 15 with L and 15 with R hemiparesis): ST 2–5 h/d, 5 d/w for 21 d	FAT MSS FIR	MT was not superior to ST in terms of improving motor recovery of the upper arm. Yet, MT resulted in greater improvement in the domain of self-care independence (assessed by FIR), but only for the subgroup with R hemiparesis.
Thieme et al. Germany 2013 [36]	<3 mo Mean 45 d Both types	60	MTG1 (*n* = 18): 30-min sessions of individual MT (bilateral movements), 3–5 d/w for 5 w (20 sessions in total) + ST MTG2 (*n* = 21): Equivalent 30-min sessions of group MT + ST CG (*n* = 21): Equivalent 30-min sessions of group sham therapy (wooden board) + ST	FMA-UE ARAT BI MAS	MT, administered either as an individual or group intervention, was not found to be more effective than control therapy in terms of improving motor function, spasticity or independence in activities of daily living.

Abbreviations: ARAT = action research arm test; BI = Barthel index; CG = control group; d = day(s); FAT = Frenchay arm test; FIM = functional independence measure; FIR = functional index ‘’Repty’’; FMA-UE = Fugl–Meyer assessment test for the upper extremity; h = hour; L = left; MAS = modified Ashworth scale; min = minutes; MI-UE = motricity index for the upper extremity; mo = month(s); MSS = motor status scale; MT = mirror therapy; MTG = mirror therapy group; NDT = neurodevelopmental treatment; R = right; ST = standard therapy; SULCS = stroke upper limb capacity scale; w = week(s); WMFT = Wolf motor function test.

**Table 3:** Studies involving the application of mirror therapy in both early and late subacute phases of stroke.

Author Country Year	Stroke Duration and Type	Number of Patients	Study Design	Assessment Tools	Major Finding
Gurbuz et al. Turkey 2016 [38]	<6 mo Mean 44.3 d Both types	31	MTG (*n* = 16): 20 min MT (unilateral movements) + 60–120 min ST, 5 d/w for 4 w CG (*n* = 15): Equivalent sham therapy (opaque surface of the mirror) + ST	FMA-UE BSSR FIM	MT was more effective than control therapy in the improvement of the paretic limb’s motor function based on FMA-UE.
Lim et al. South Korea 2016 [39]	<6 mo Mean 51.6 d Both types	60	MTG (*n* = 30): 20 min MT (bilateral movements), 5 d/w for 4 w + ST CG (*n* = 30): Equivalent sham therapy (wooden board) + ST	FMA-UE BSSR MBI	Patients receiving task-oriented MT had greater improvement in FMA-UE and MBI compared to CG.
Madhoun et al. China 2020 [40]	<6 mo Mean 3.9 mo Both types	30	MTG (*n* = 15): 25 min MT (unilateral movements), 7 d/w for 25 d + ST CG (*n* = 15): 25 min OT + ST	FMA-UE BSSR MAS MBI	Compared to OT, MT resulted in greater improvement in motor function, as assessed by FMA-UE, but not by BSSR.
Samuelkamaleshkumar et al. India 2014 [12]	<6 mo Mean 4 w Both types	20	MTG (*n* = 10): 30-min sessions of MT twice daily (bilateral movements), 5 d/w for 3 w + 360 min ST CG (*n* = 10): 360 min ST	FMA-UE BSSR BBT MAS	MT led to improvement in motor control (FMA-UE), motor recovery (BSSR) and gross manual dexterity (BTT), but not in spasticity (MAS).
Lee et al. South Korea 2012 [42]	<6 mo Mean 3.55 mo Type not defined	26	MTG (*n* = 13): 25-min sessions of MT twice daily (bilateral movements), 5 d/w for 4 w + 105 min ST CG (*n* = 13): 105 min ST	FMA-UE BSSR MFT	Compared to ST, MT was significantly more effective in enhancing motor recovery, as evidenced by all 3 assessment tools.
Bae et al. South Korea 2012 [44]	<6 mo Mean 4.6 mo Both types	20	MTG (*n* = 10): 30 min MT (bilateral movements), 5 d/w for 4 w + ST CG (*n* = 10): Equivalent sham therapy (no mirror, unilateral movements) + ST	MFT	The application of MT led to greater improvement in the motor function of the paretic upper limb and to increased activation of the cortical motor areas.
Wen et al. China 2022 [45]	<6 mo Mean 30.5 d Both types	52	MTG (*n* = 25): 30 min MT (bilateral movements), 6 d/w for 3 w + 30 min ST CG (*n* = 27): 30 min ST	FMA-UE ARAT LIADL	Compared to CG, MTG showed significantly greater improvements in FMA-UE and LIADL, but not in ARAT.
Arfianti et al. Indonesia 2022 [47]	3 w–6 mo Mean 5 w Both types	18	MTG (*n* = 9): 20 min MT (bilateral movements), 2 d/w for 5 w + 20 min ST CG (*n* = 9): 20 min ST, 2 d/w for 5 w	BSSR FIM	Patients in the MTG showed statistically greater improvement in both BSSR and FIM scales compared to the CG.
Cacchio et al. Italy 2009 [48]	<6 mo Mean 5 mo Both types	48	MTG (*n* = 24): 30 min MT (unilateral movements) for first 2 w and 1 h MT for next 2 w per session on top of 1 h ST, 5 d/w for 4 w in total CG (*n* = 24): Equivalent sham therapy (covered mirror) + 1 h ST	WMFT MAL	Patients in the MTG showed statistically more significant improvements than CG in WMFT and in the quality of movement scale of MAL, both post-treatment and at 6-month follow-up.
Rehani et al. India 2015 [49]	1–6 mo Mean 83.4 d Both types	12	MTG (*n* = 6): 60 min MT (bilateral movements), 6 d/w for 4 w + ST CG (*n* = 6): 60 min MRP, 6 d/w for 4 w + ST	CAHAI	MT was not found to be superior to MRP in terms of promoting recovery of the paretic hand’s motor function.

Abbreviations: ARAT = action research arm test; BBT = box and block test; BSSR = Brunnstrom stages of stroke recovery; CAHAI = Chedoke arm and hand activity inventory; CG = control group; d = day(s); FIM = functional independence measure; FMA-UE = Fugl–Meyer assessment test for the upper extremity; h = hour; LIADL = Lawton instrumental activities of daily living scale; MAL = motor activity log; MAS = modified Ashworth scale; MBI = modified Barthel index; MFT = manual function test; min = minutes; mo = month(s); MRP = motor relearning program; MT = mirror therapy; MTG = mirror therapy group; OT = occupational therapy; ST = standard therapy; w = week(s); WMFT = Wolf motor function test.

**Table 4:** Studies in the chronic phase of stroke demonstrating positive effects of mirror therapy.

Author Country Year	Stroke Duration and Type	Number of Patients	Study Design	Assessment Tools	Major Finding
Michielsen et al. Netherlands 2011 [51]	>1 yr Mean 3.9 yr Both types	40	MTG (*n* = 20): 60 min MT (bilateral movements), 6 d/w (5 d at home and 1 d at rehab center) for 6 w CG (*n* = 20): Equivalent sham therapy (no mirror)	FMA-UE ARAT Jamar dynamometer Tardieu scale ABILHAND Stroke-ULAM EQ-5D	Compared to the CG, patients in the MTG showed greater improvement only in FMA-UE at 6 w, which however was not statistically significant at 6 mo. At 6 w, fMRI showed changes in the activation pattern of the primary motor cortex only in the MTG.
Park et al. South Korea 2015 [52]	>6 mo Mean 20.9 mo Both types	30	MTG (*n* = 15): 30 min MT (bilateral movements), 5 d/w for 4 w + OT CG (*n* = 15): Equivalent sham therapy (opaque mirror) + OT	FMA-UE BBT FIM	MT led to statistically more significant improvements in all assessment tools compared to sham therapy.
Kim et al. South Korea 2016 [53]	>6 mo Both types	25	MTG (*n* = 12): 30 min task-oriented MT (unilateral movements), 5 d/w for 4 w CG (*n* = 13): 30 min task-based ST	FMA-UE ARAT BBT FIM	MT resulted in greater improvement in motor function/performance and in ADL with all assessment tools.
Chinnavan et al. Malaysia 2020 [54]	>6 mo Type not defined	25	MTG (*n* = 13): 15 min MT (unilateral movements) + 30 min ST, 3 d/w for 6 w CG (*n* = 12): 45 min ST	FMA-UE FIM	The MTG experienced statistically more significant improvements in FMA-UE and FIM compared to the CG.
Wu et al. Taiwan 2013 [55]	>6 mo Mean 20.6 mo Both types	33	MTG (*n* = 16): 60 min MT (bilateral movements), 5 d/w for 4 w + 30 min task-oriented ST CG (*n* = 17): 90 min task-oriented ST	FMA-UE MAL ABILHAND Kinematic analysis	MT led to greater improvements in motor function of the distal (but not the proximal) upper limb, as well as in certain kinematic parameters.
Arya et al. India 2013 [56]	>6 mo Mean 34.45 mo Both types	13	MTG (*n* = 13): 30 min task-based MT (unilateral movements), 4 d/w for 4 w + 30 min OT No control group.	FMA-UE BSSR	The application of MT led to statistically significant improvement in the FMA-UE scores only for the wrist and hand, compared to pre-treatment values.
Arya et al. India 2015 [57]	>6 mo Mean 12.5 mo Both types	33	MTG (*n* = 17): 45 min task-based MT (unilateral movements), 5 d/w for 8 w + 45 min OT CG (*n* = 16): 90 min OT	FMA-UE BSSR	Compared to CG, MTG showed significantly greater motor recovery of the wrist and hand as per the FMA-UE tool.
Fong et al. Hong Kong 2019 [58]	>6 mo Mean 26.8 mo Both types	101	MTG (*n* = 51): 30-min sessions of MT (bilateral movements), 2 d/w for 6 w CG (*n* = 50): Equivalent therapy with bilateral arm training (no mirror)	WMFT FMA-UE ARAT	Patients in the MTG showed better results only in the hand subscore of the FMA-UE tool compared to patients in the CG.
Kim et al. South Korea 2015 [59]	>6 mo Mean 7.29 mo Both types	14	MTG (*n* = 14): 30 min MT (bilateral movements), 5 d/w for 4 w No control group.	MFT	MT resulted in statistically significant improvements in the MFT items related to fine and gross dexterity of the hand.
Shaker et al. Egypt 2020 [60]	>6 mo Mean 22 mo Ischemic type	30	MTG (*n* = 15): 25 min MT (bilateral movements), 3 d/w for 8 w + 15 min PT CG (*n* = 15): Equivalent sham therapy (no mirror) + 15 min PT	JTHFT Jamar dynamometer Electronic goniometer	MT resulted in greater improvement of hand motor function with all assessment tools.
Cacchio et al. Italy 2009 [61]	Median 14 mo (range 7–21) Both types	24	MTG (*n* = 8): 30 min MT (unilateral movements) for 4 w CG1 (*n* = 8): 30 min sham therapy (covered mirror) for 4 w CG2 (*n* = 8): Mental imagery for 4 w	WMFT	MT enhanced the paretic arm’s motor function in patients with chronic stroke and CRPS1, unlike sham MT or mental imagery therapy.
Saha et al. India 2021 [62]	>1 yr Mean 13.37 mo Both types	30	MTG (*n* = 15): 30 min MT (bilateral movements), 5 d/w for 4 w CG (*n* = 15): Equivalent sham therapy (no mirror)	FIM	MT enhanced ADL to a greater extent compared to sham therapy. Improvement was maintained at 2-week follow-up.
Paik et al. South Korea 2014 [63]	>6 mo Mean 30.75 mo Both types	4	MTG1 (*n* = 2): 30 min simple MT (5 simple unilateral movements), 15 sessions + ST MTG2 (*n* = 2): 30 min task-oriented MT (unilateral ADL tasks), 15 sessions + ST	FMA-UE BBT MFT (cube carry test) JTHFT (card turning test)	Motor function improved in both MT groups. MTG2 continued to improve after MT cessation, whereas MTG1 started to decline. Task-oriented MT was more effective than simple MT.
Oliveira et al. Brazil 2018 [64]	>1 yr Type not defined	21	MTG (*n* = 7): MT of undefined duration (bilateral movements, 2 sets of 10 repetitions), 3 d/w for 4 w VTG (*n* = 7): 15 min vibration therapy CG (*n* = 7): conventional PT	WMFT JTHFT Rivermead mobility index	Compared to the CG, both MTG and VTG showed statistically more significant improvements in motor function with all assessment tools.

Abbreviations: ADL = activities of daily living; ARAT = action research arm test; BBT = box and block test; BSSR = Brunnstrom stages of stroke recovery; CG = control group; CRPS1 = complex regional pain syndrome type 1; d = day(s); EQ-5D = EuroQol-5 dimension questionnaire; FIM = functional independence measure; FMA-UE = Fugl–Meyer assessment test for the upper extremity; fMRI = functional magnetic resonance imaging; JTHFT = Jebsen–Taylor hand function test; MAL = motor activity log; MFT = manual function test; min = minutes; mo = month(s); MT = mirror therapy; MTG = mirror therapy group; OT = occupational therapy; PT = physiotherapy; ST = standard therapy; Stroke-ULAM = stroke upper limb activity monitor; VTG = vibration therapy group; w = week(s); WMFT = Wolf motor function test; yr = year(s).

**Table 5:** Studies in the chronic phase of stroke demonstrating neutral effects of mirror therapy.

Author Country Year	Stroke Duration and Type	Number of Patients	Study Design	Assessment Tools	Major Finding
Colomer et al. Spain 2016 [65]	>6 mo Mean 18.37 mo Both types	31	MTG (*n* = 15): 45 min MT (unilateral movements), 3 d/w for 8 w + 60 min PT CG (*n* = 16): 45 min passive mobilization, 3 d/w for 8 w + 60 min PT	WMFT FMA-UE	There was no statistically significant difference between the MTG and the CG in terms of motor function improvement.
Rodrigues et al. Brazil 2016 [66]	>6 mo Mean 34.8 mo Ischemic type	16	MTG (*n* = 8): 60 min MT (bilateral movements), 3 d/w for 4 w CG (*n* = 8): Equivalent sham therapy (covered mirror)	TEMPA FMA-UE	Both MTG and CG demonstrated similar extent of improvement in motor function and activity level of the paretic upper limb.
Li et al. Taiwan 2019 [68]	>6 mo Mean 53 mo Both types	23	MTG (*n* = 12): MT (bilateral movements), 90 min at hospital (3 d/w) and 30–40 min at home (5 d/w) for 4 w CG (*n* = 11): Equivalent sham therapy (no mirror)	FMA-UE CAHAI MAL Stroke Impact Scale	No significant difference was found in the improvement of motor function between the two groups. Yet, the MTG showed greater improvements in QOL.
de Medeiros et al. Brazil 2014 [69]	>6 mo Mean 5 yr Ischemic type	6	MTG1 (*n* = 3), 30 min task-based MT (bilateral movements), 3 d/w for 5 w MTG2 (*n* = 3), 30 min MT based on simple bilateral movements, 3 d/w for 5 w	FMA-UE FIM MAS	Neither MT group showed any significant differences pre- and post-treatment. Also, no differences were found in the comparison between the 2 groups.
Ehrensberger et al. Ireland 2019 [70]	>6 mo Mean 81.9 mo Both types	32	MTG (*n* = 17): 20 min MT combined with isometric exercises (unilateral movements), 3 d/w for 4 w CG (*n* = 15): 20 min isometric strength training of the unaffected upper limb	CAHAI ABILHAND MAS LHS Biodex dynamometer	MT combined with isometric exercises was not found to be more effective when compared to isometric strength training alone.
Selles et al. Netherlands 2014 [71]	>6 mo Mean 31.6 mo Type not defined	93	MTG1 (*n* = 17): MT with bilateral movements (reaching task exercise) MTG2 (*n* = 20): MT with unilateral movements of the unaffected arm CG1 (*n* = 18): sham MT (opaque screen) with bilateral movements CG2 (*n* = 21): unilateral movements of the unaffected arm under direct vision CG3 (*n* = 17): unilateral movements of the affected arm under direct vision	Kinematic analysis FMA-UE	CG3 showed the greatest improvement in terms of time required to complete the movement, followed by MTG2, whereas the remaining groups showed the smallest improvements. The therapeutic schemes which involved bilateral training had relatively small effects on movement time.

Abbreviations: CAHAI = Chedoke arm and hand activity inventory; CG = control group; d = day(s); FIM = functional independence measure; FMA-UE = Fugl–Meyer assessment test for the upper extremity; LHS = London handicap scale; MAL = motor activity log; MAS = modified Ashworth scale; min = minutes; mo = month(s); MT = mirror therapy; MTG = mirror therapy group; PT = physiotherapy; TEMPA = test d’évaluation des membres supérieurs des personnes âgées (Upper extremity performance test for the elderly); w = week(s); WMFT = Wolf motor function test.

**Table 6:** Studies involving the application of mirror therapy in more than one phase of stroke.

Author Country Year	Stroke Duration and Type	Number of Patients	Study Design	Assessment Tools	Major Finding
Waghavkar et al. India 2015 [72]	2–45 d Both types	11	MTG (*n* = 11): 50 min MT with 20 min warm-up and 30 min MT training (bilateral movements) + ST, 4 d/w for 4 w No control group	FMA-UE WMFT	MT led to significant improvement in the motor function of the distal arm, based on the hand subsections of both assessment tools.
Yavuzer et al. Turkey 2008 [73]	<1 yr Mean 5.5 mo Both types	36	MTG (*n* = 17): 30 min MT (bilateral movements), 5 d/w for 4 w + 2–5 h ST CG (*n* = 19): Equivalent sham therapy (opaque surface of mirror) + 2–5 h ST	BSSR MAS FIM	At 4 w and at 6-mo follow-up, the MTG had greater improvement in BSSR and FIM, but not in MAS, compared to the CG.
Tripathi et al. India 2016 [74]	<1 yr Mean 4.48 mo Both types	35	MTG (*n* = 18): 30 min MT (bilateral movements), 5 d/w for 4 w + 2–3 h (at hospital) and 1 h (at home) ST, 6 d/w for 4 w CG (*n* = 17): Equivalent sham therapy (opaque surface of mirror) + ST	BSSR ARAT MAS FIM	After 4 w, MT resulted in greater improvement in BSSR, ARAT and FIM, but not in MAS. At 6-mo follow-up, only FIM score continued to improve significantly.
Rajappan et al. Malaysia 2015 [75]	2 mo–1 yr Mean 5 mo Both types	30	MTG (*n* = 15): 30 min MT (bilateral movements), 5 d/w for 4 w + 1 h ST CG (*n* = 15): Equivalent sham therapy (opaque surface of mirror) + 1 h ST	FMA-UE UEFI	MT led to significantly greater improvement in motor function (FMA-UE) and in functional performance of ADL (UEFI).
Pervane Vural et al. Turkey 2016 [76]	<12 mo 60–240 d Both types	30	MTG (*n* = 15): 30 min MT (bilateral movements), 5 d/w for 4 w + 2–4 h ST CG (*n* = 15): 2–4 h ST	FMA-UE BSSR MAS FIM	Compared to the CG, the MTG showed significantly greater improvement in the wrist and hand sections of FMA-UE and in the motor items of FIM. No improvement was observed in MAS.
Champaiboon et al. Thailand 2017 [77]	>12 w 3–72 mo Both types	40	MTG (*n* = 20): 30 min MT (bilateral movements), 5 d/w for 2 w + 6 h ST, 5 d/w for 8 w CG (*n* = 20): Equivalent sham therapy (opaque surface of mirror), 5 d/w for 2 w + 6 h ST, 5 d/w for 8 w	BSSR MAS BI MotorAS Pinch strength	Compared to the CG, the MTG showed significantly greater improvement only in the BSSR scale for the hand at 2 w, which however was not maintained at 4-w, 8-w and 12-w follow-up.
Khandare et al. India 2013 [78]	2 mo–1 yr Mean 193 d Both types	37	MTG1 (*n* = 12): 30 min MT (unilateral movements) + PT, 5 d/w for 4 w MTG2 (*n* = 13): 30 min MT (unilateral movements) + 30 min task-based training of paretic arm + PT, 5 d/w for 4 w TBTG (*n* = 12): 30 min task-based training of paretic arm + PT, 5 d/w for 4 w	FMA-UE ARAT VCG	All groups showed improvement in ARAT and FMA-UE. However, the largest improvement was seen in MTG2 (combined MT with task-based training), followed by TBTG (task-based training), whereas MTG1 (MT alone) had the smallest improvement.
Jan et al. Pakistan 2019 [79]	Undetermined stroke duration Both types	66	MTG (*n* = 33): 2 h MT, 3 d/w for 6 w + 20 min ST MRPG (*n* = 33): 2 h MRP, 3 d/w for 6 w	MotorAS	Patients in the MRPG showed statistically greater improvement in the upper extremity subscales of the MotorAS compared to the MTG.
Zhang et al. China 2021 [80]	Undetermined inclusion criteria for stroke duration Mean 30.5 d Type not defined	60	MTG (*n* = 30): 60 min MT (unilateral movements), 5 d/w for 4 w + 30 min OT CG (*n* = 30): 60 min OT (2 sessions of 30 min daily), 5 d/w for 4 w	FMA-UE MBI	Patients in the MTG showed statistically more significant improvements in motor function and ADL performance than the CG.

Abbreviations: ADL = activities of daily living; ARAT = action research arm test; BI = Barthel index; BSSR = Brunnstrom stages of stroke recovery; CG = control group; d = day(s); FIM = functional independence measure; FMA-UE = Fugl–Meyer assessment test for the upper extremity; h = hour(s); MAS = modified Ashworth scale; MBI = modified Barthel index; min = minutes; mo = month(s); MotorAS = motor assessment scale; MRP = motor relearning programme; MRPG = motor relearning programme group; MT = mirror therapy; MTG = mirror therapy group; OT = occupational therapy; PT = physiotherapy; ST = standard therapy; TBTG = task-based training group; UEFI = upper extremity functional index; VCG = voluntary control grading scale; w = week(s); WMFT = Wolf motor function test; yr = year(s).

## Data Availability

Not applicable.
